# Population structure reveals clade- and region-specific dissemination of macrolide-resistant *Mycoplasma pneumoniae*

**DOI:** 10.3389/fcimb.2026.1891292

**Published:** 2026-08-10

**Authors:** Takeshi Yamamoto, Miki Okuno, Yuki Hoshiko, Shunsuke Iriguchi, Yoshitoshi Ogura, Tomohiro Oishi

**Affiliations:** 1 Division of Microbiology, Department of Infectious Medicine, Kurume University School of Medicine, Kurume, Japan; 2 Department of Health Science, School of Allied Health Sciences, Kitasato University, Sagamihara, Japan; 3 Department of Clinical Infectious Diseases, Kawasaki Medical School, Kurashiki, Japan

**Keywords:** clonal expansion, macrolide resistance, *Mycoplasma pneumoniae*, phylogenetic analysis, population structure

## Abstract

**Objectives:**

Macrolide-resistant *Mycoplasma pneumoniae* (MRMP) has become highly prevalent in East Asia since the early 2000s, whereas its prevalence remains relatively low in many other regions. This striking geographic disparity has often been attributed to the expansion of a limited number of resistant lineages. However, the population-structural basis of this regional pattern remains incompletely understood. To address this, we analyzed whole-genome sequences of 613 *M. pneumoniae* strains, including 86 strains isolated in Japan and 527 publicly available genomes, to investigate the relationships among phylogenetic structure, geographic distribution, and macrolide resistance.

**Results:**

Macrolide resistance was identified in 61.5% (377/613) of strains, and 94.2% of resistant strains carried the A2063G mutation in the 23S rRNA gene. Resistant strains were strongly concentrated in East Asia (70.5% vs 13.4% in non–East Asian regions), with resistance rates varying across countries, ranging from 30.4% in Japan to 99.0% in China. This pattern cannot be fully explained by the expansion of the well-characterized resistant clade T1-3R alone, but is more likely attributable to the emergence of resistance in multiple genetic backgrounds. Notably, in T1–2 and T2-2, resistance rates among East Asian strains were 66.0% and 45.4%, respectively, whereas no resistant strains were identified in non–East Asian populations, highlighting regional disparities within the same clonal backgrounds. Phylogenetic network analysis of these clades further suggested that resistant variants did not arise sporadically but appeared to reflect mutation acquisition followed by regional clonal expansion. In contrast to the geographically restricted expansion of resistant clones, macrolide-susceptible strains from different regions occasionally showed close genetic relatedness within 10 SNPs, suggesting that cross-regional dissemination of *M. pneumoniae* strains can occur.

**Conclusions:**

These findings suggest that the high prevalence of MRMP in East Asia cannot be explained solely by the expansion of a single resistant lineage. Instead, multiple resistant clones appear to have emerged and expanded within several clades that are prevalent in East Asia. This population-structured view of MRMP dissemination provides a framework for understanding regional differences in resistance dynamics and underscores the importance of continued genome-based surveillance to monitor the emergence and regional spread of resistant clones.

## 1 Introduction

*Mycoplasma pneumoniae* is a human-pathogenic mycoplasma that primarily causes respiratory tract infections, including bronchitis and pneumonia. It is recognized as an important etiological agent of community-acquired pneumonia, accounting for up to 40% of all cases (Jacobs et al., 2015; Waites et al., 2017; Parrott et al., 2016). *M. pneumoniae* infections occur most frequently among children and young adults, particularly those aged 5–17 years, with a higher burden observed in pediatric populations (Waites et al., 2017; Parrott et al., 2016; Centers for Disease Control and Prevention, 2024). Consequently, *M. pneumoniae* infection is regarded as a clinically important infectious disease in the field of pediatric medicine.

*M. pneumoniae* lacks a peptidoglycan-containing cell wall and therefore exhibits intrinsic resistance to β-lactam antibiotics. Accordingly, macrolide antibiotics, which can be safely administered to children, have been widely used as first-line agents for the treatment of *M. pneumoniae* infections. However, since the early 2000s, the emergence and global increase of macrolide-resistant *M. pneumoniae* (MRMP) have been reported, and clinically refractory cases in which antimicrobial chemotherapy is ineffective have emerged as a significant clinical concern (Kim et al., 2022; Wang et al., 2022; Meyer et al., 2016). The prevalence of MRMP varies markedly by geographic region and is known to be particularly high in East Asia, including Japan, China, South Korea, and Taiwan (Liu et al., 2024; Wang et al., 2024; Wu et al., 2024; Kenri et al., 2020). Indeed, during the global outbreaks of *M. pneumoniae* infections in the 2010s, a large number of MRMP cases were reported from this region, suggesting the presence of region-specific factors that may contribute to the dissemination of resistant strains.

*M. pneumoniae* has traditionally been classified into type 1 and type 2 lineages (Su et al., 1990; Ruland et al., 1994); however, recent analyses have further subdivided type 1 strains into T1-1, T1-2, T1-3, and T1-3R, and type 2 strains into T2–1 and T2-2 (Kenri et al., 2023; Hsieh et al., 2022; Kubota et al., 2025; Wang et al., 2025b). Among these, the T1-3R clade predominantly consists of macrolide-resistant strains, has been detected at a high frequency in East Asia, and has been proposed as a major contributor to the elevated prevalence of macrolide resistance in this region (Kenri et al., 2023; Sun et al., 2025; Fang et al., 2026). However, much of the existing evidence is based on limited genotyping approaches, such as multilocus sequence typing (MLST), multilocus variable-number tandem-repeat analysis (MLVA), and P1 typing (Kenri et al., 2020; Brown et al., 2015; Ando et al., 2018), and the detailed phylogenetic relationships among strains as well as their patterns of regional and interregional dissemination remain incompletely understood at a genome-wide scale.

In this study, we performed a comprehensive phylogenomic analysis using genome sequences of *M. pneumoniae* strains retrieved from public databases, together with strains isolated in Japan, to elucidate the genetic relatedness of strains across regions and to characterize the distribution of clades and macrolide-resistant strains. Our results suggest that the high prevalence of macrolide resistance in East Asia cannot be explained solely by the circulation of a single resistant clade, as previously proposed, but is also associated with the expansion of resistant lineages within multiple clades that are predominant in this region. These findings highlight the importance of comprehensive genome-based analyses for understanding the mechanisms underlying regional differences in the epidemiology of *M. pneumoniae*.

## 2 Materials and methods

### 2.1 Sample collection and whole-genome sequencing

Nasopharyngeal swab specimens were collected from 86 pediatric patients with *M. pneumoniae* infection between 2009 and 2020, mainly in the Chugoku region of Japan, after informed consent was obtained from their parents or guardians. The study protocol was approved by the Ethics Committee of Kawasaki Medical School (Kurashiki, Japan; approval no. 3119-08). Specimens were inoculated into PPLO broth and enriched prior to DNA extraction, and genomic DNA was extracted from enriched cultures using a previously described method (Tanaka et al., 2017). Extracted DNA was stored at −80 °C until use. DNA concentrations were measured using the Quant-iT PicoGreen dsDNA Assay Kit (Thermo Fisher Scientific, Waltham, MA, USA). Sequencing libraries were prepared using the xGen DNA Library Prep EZ Kit (Integrated DNA Technologies, Coralville, IA, USA), and index sequences were added using the NEBNext Multiplex Oligos for Illumina 96 Unique Dual Index Primer Pairs (New England Biolabs, Ipswich, MA, USA). Paired-end sequencing (151 bp) was performed on the Illumina HiSeq X Ten platform.

### 2.2 Collection of publicly available genome data

Publicly available genome data of *M. pneumoniae* were retrieved from the NCBI database. We collected genome sequences for which information on the year and geographic location of isolation was available. A total of 303 assembled genomes were obtained. In addition, 582 sequence read archive (SRA) datasets were retrieved, limited to paired-end FASTQ data generated using Illumina platforms. Sequences derived from laboratory-generated mutant strains, including those produced through genetic manipulation or extensive serial passage, were excluded from the analysis. When both an assembled genome and SRA data originating from the same isolate were available, the assembled genome was preferentially used. As a result of these filtering steps, 7 assembled genomes and 102 SRA datasets were excluded, and the remaining publicly available datasets were retained for downstream analyses.

### 2.3 *de novo* assembly

*De novo* assembly was performed using Platanus_B v1.3.2 (Kajitani et al., 2020) for sequencing reads generated in this study and paired-end reads obtained from NCBI. Because the sequencing depth exceeded 100× for the majority of samples, reads were randomly subsampled to a maximum depth of 100× prior to assembly to reduce potential assembly bias and computational burden. Among the SRA datasets, six samples contained a substantial proportion of reads derived from organisms other than *M. pneumoniae* and could not be assembled reliably; these samples were therefore excluded from further analyses.

### 2.4 Draft genome quality assessment

Quality assessment of draft genomes was performed using CheckM v1.2.0 (Parks et al., 2015). Genomes with an estimated completeness of <97% or contamination of >3% were excluded from downstream analyses. In addition, draft genomes with an N50 value of less than 5,000 bp were also excluded. All genomes newly sequenced in this study met these quality criteria. For publicly available data, draft genomes derived from the same laboratory strain were sometimes registered multiple times. In such cases, the genome with higher assembly quality and the more recent submission year was retained. After quality filtering, a total of 613 genomes were included in the final dataset, comprising 245 genomes derived from SRA data, 282 assembled genomes from public databases, and 86 genomes newly generated in this study.

### 2.5 Genome annotation

Genome annotation was performed using Prokka v1.14.6 (Seemann, 2014). Because *M. pneumoniae* differs from typical eubacteria in its genetic code, in which the UGA codon encodes tryptophan rather than serving as a stop codon, annotation was conducted using the option --gcode 4.

### 2.6 Core-genome phylogenetic analysis

Phylogenetic relationships among strains were inferred based on single-nucleotide polymorphism (SNP) sites identified within the core genome. The core genome was defined using Roary v3.13.3 (Page et al., 2015) with a BLASTP identity threshold of 92%. Genes present in ≥99% of the analyzed genomes (Roary default setting) were considered core genes, and Roary was used to generate a concatenated alignment of these genes. SNP sites were subsequently extracted from the core-genome alignment using SNP-sites v2.5.1 (Page et al., 2016), and the resulting SNP alignment was used for phylogenetic inference. No explicit filtering of recombinant regions was performed prior to phylogenetic inference. Maximum-likelihood phylogenetic inference was performed using RAxML-NG v1.0.1 (Kozlov et al., 2019) under the GTR+G4 substitution model, with 100 bootstrap replicates (--bs-trees 100). The resulting phylogenetic tree was visualized using iTOL v6.6 (Letunic and Bork, 2021).

### 2.7 Detection of macrolide resistance–associated SNPs

Single-nucleotide polymorphisms in the 23S rRNA gene associated with macrolide resistance in *M. pneumoniae* were identified by sequence comparison with the macrolide-susceptible reference strain FH. The 23S rRNA gene sequences from each strain were extracted and aligned using MAFFT v7.487 (Katoh and Standley, 2013), and SNPs were subsequently identified using SNP-sites. In this study, strains harboring mutations at adenine positions 2063 and 2064, or cytosine position 2617 in the 23S rRNA gene were defined as macrolide-resistant strains.

### 2.8 MLST analysis

Multilocus sequence typing (MLST) was performed using the reference allele sequences of eight housekeeping genes available in the pubMLST database (Jolley et al., 2018). Allele types were assigned based on sequence similarity identified by BLASTN, and MLST types were determined according to the corresponding allelic profiles. In a subset of strains, a single-nucleotide mismatch was observed in one of the target genes. In such cases, the closest matching MLST type was assigned, with the deviation explicitly annotated.

### 2.9 Clade classification

Clade classification of *M. pneumoniae* was performed according to the method described by Kenri et al. (2023). Type 1 lineage strains were classified into four clades (T1-1, T1-2, T1-3, and T1-3R), while type 2 lineage strains were classified into two clades (T2–1 and T2-2). For Type 1 lineage strains, classification was based on single-nucleotide polymorphisms (SNPs) located in MPN678, MPN607, and MPN295. These SNPs are located within ApoI restriction enzyme recognition sites, allowing clade assignment based on the corresponding restriction fragment patterns. In contrast, Type 2 lineage strains were classified based on phylogenetic clustering, using MLST types of reference strains with known clade assignments as a guide.

### 2.10 Phylogenetic network analysis

The genetic relationships among strains originating from different geographic regions within the same clade were visualized using phylogenetic network analysis with POPART v1.7 (Leigh and Bryant, 2015). An alignment constructed from core genome–derived SNP sites was used as input data, and the network was inferred using the median-joining method (Bandelt et al., 1999).

### 2.11 Statistical analysis

Differences between groups were evaluated using Fisher’s exact test implemented in R (v4.4.1; fisher.test). For each analysis, one group was compared with all remaining groups combined. The resulting P values were adjusted for multiple testing using the Benjamini–Hochberg method.

## 3 Results

### 3.1 Dataset composition and geographic distribution of *M. pneumoniae* strains

**Figure 1:**
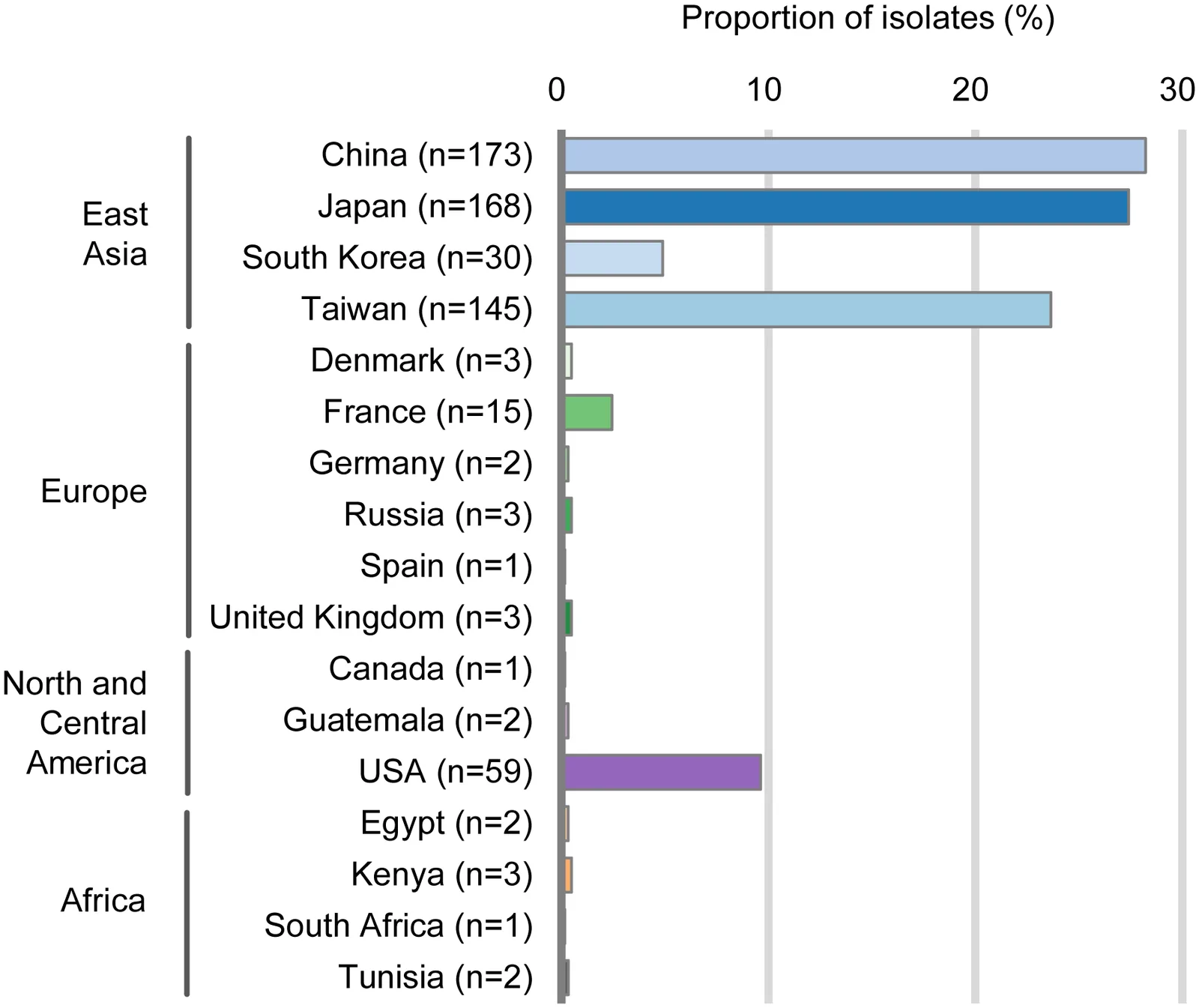
Geographic distribution of the 613 *M. pneumoniae* strains. The proportional distribution of strains by geographic origin is shown as a percentage bar chart. The absolute number of strains is indicated in parentheses.

To elucidate the basis of the marked geographic differences in the proportion of macrolide-resistant strains in *M. pneumoniae*, we analyzed a total of 613 *M. pneumoniae* genome sequences, including 86 strains isolated in Japan in this study and 527 strains retrieved from public genome databases (**Supplementary Table 1**). The geographic origins of the analyzed strains were unevenly distributed (**Figure 1**). To provide an overview of the sampling framework, the numbers of strains stratified by country and year of isolation are summarized in **Supplementary Table 2**. Strains from East Asia, including Japan, China, South Korea, and Taiwan, accounted for 84.2% of the total dataset, whereas strains from Europe, North and Central America, and Africa were limited in number. Nevertheless, strains from these regions were included to enable comparative analyses across geographic regions. Notably, more than 10 strains were available from several countries, including Japan, China, South Korea, and Taiwan in East Asia, as well as the United States and France. The latter two largely represented North and Central America and Europe, respectively, indicating that the dataset was partly driven by a limited number of countries within each region. Given this marked imbalance in geographic representation, together with the known regional disparity in macrolide resistance, subsequent analyses primarily focused on comparisons between East Asian and non–East Asian regions.

### 3.2 Overall prevalence and regional differences of macrolide-resistant strains

**Figure 2:**
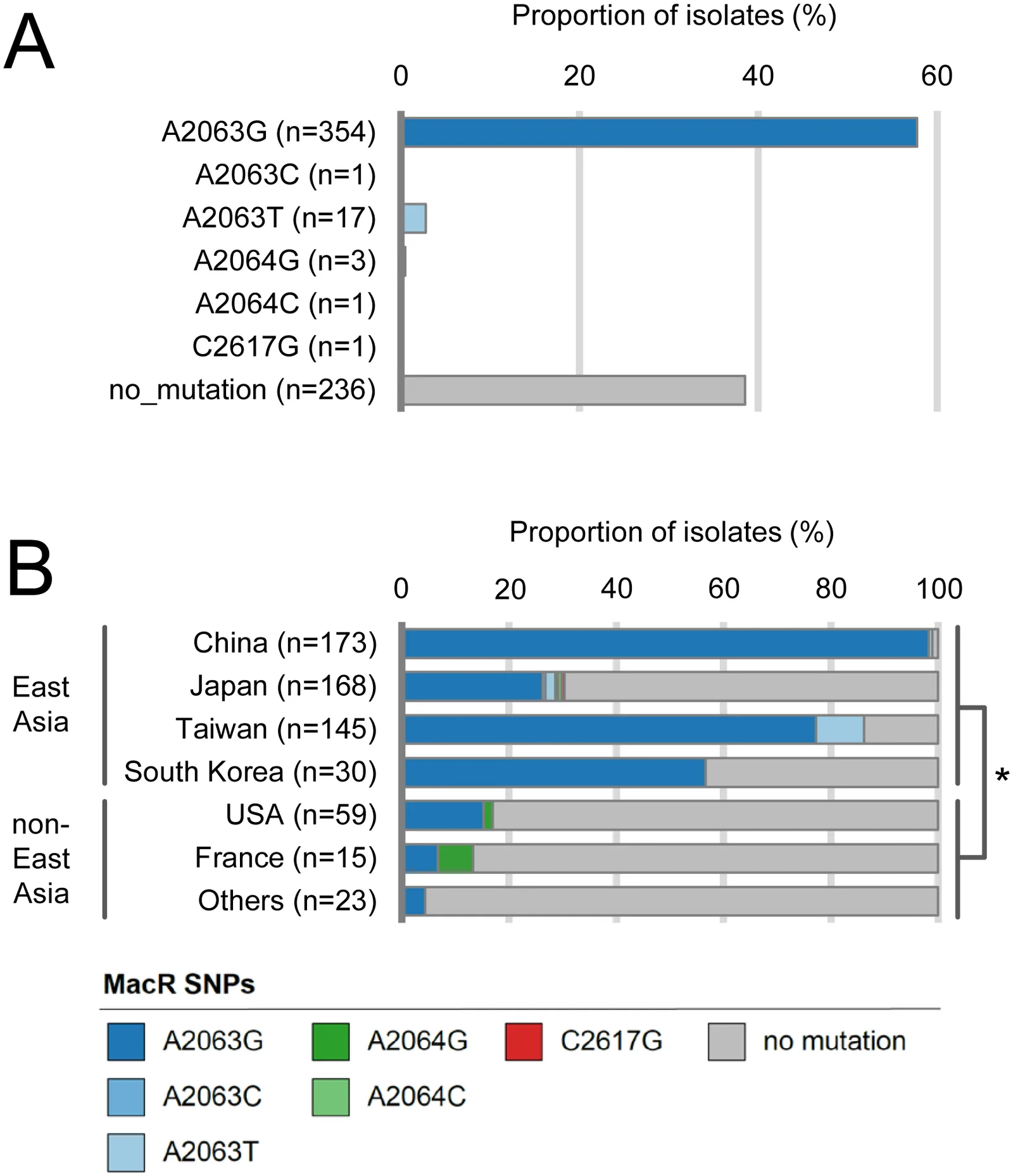
Distribution of macrolide resistance–associated 23S rRNA mutations across geographic regions. **(A)** The proportion of strains carrying macrolide resistance–associated SNPs in the 23S rRNA gene is shown as a percentage bar chart. The absolute number of strains carrying each SNP is indicated in parentheses. **(B)** The proportion of strains carrying macrolide resistance–associated SNPs within each geographic origin is shown as a 100% stacked bar chart. The number of strains from each region is indicated in parentheses. Geographic origins with fewer than 10 strains were grouped together and presented as “Others.” For statistical analysis, strains were categorized as originating from East Asia or non–East Asian regions (including the United States, France, and “Others”), and differences in proportions were assessed using Fisher’s exact test with false discovery rate (FDR) correction. * indicates FDR-adjusted *P* < 0.05.

To characterize the prevalence and regional distribution of macrolide resistance, we examined the presence of resistance-associated mutations in the dataset. A total of 377 of the 613 analyzed *M. pneumoniae* strains harbored such mutations, corresponding to 61.5% of strains. Among these resistance-associated mutations, the vast majority consisted of the A2063G substitution in the 23S rRNA gene, which was detected in 93.9% (354/377) of MRMP strains. Other mutations, including substitutions at positions 2063 and 2064 (e.g., A2063C, A2063T, A2064G, and A2064C) as well as C2617G, were observed only sporadically (**Figure 2A**). When examined by geographic origin, notable differences in MRMP prevalence were observed. Although strains from non–East Asian regions were limited in number and unevenly distributed across countries, comparison revealed a markedly higher resistance rate in East Asia (70.5%) than in non–East Asian regions (13.4%) (**Figure 2B**). Within East Asia, resistance rates varied substantially across countries, with China showing an extremely high prevalence (99.0%), whereas Japan exhibited a comparatively lower rate (30.4%). However, even in Japan, the resistance rate remained considerably higher than that observed in non–East Asian regions. These findings are consistent with previously reported regional variation in MRMP prevalence.

### 3.3 Phylogenetic overview of *M. pneumoniae* strains

**Figure 3:**
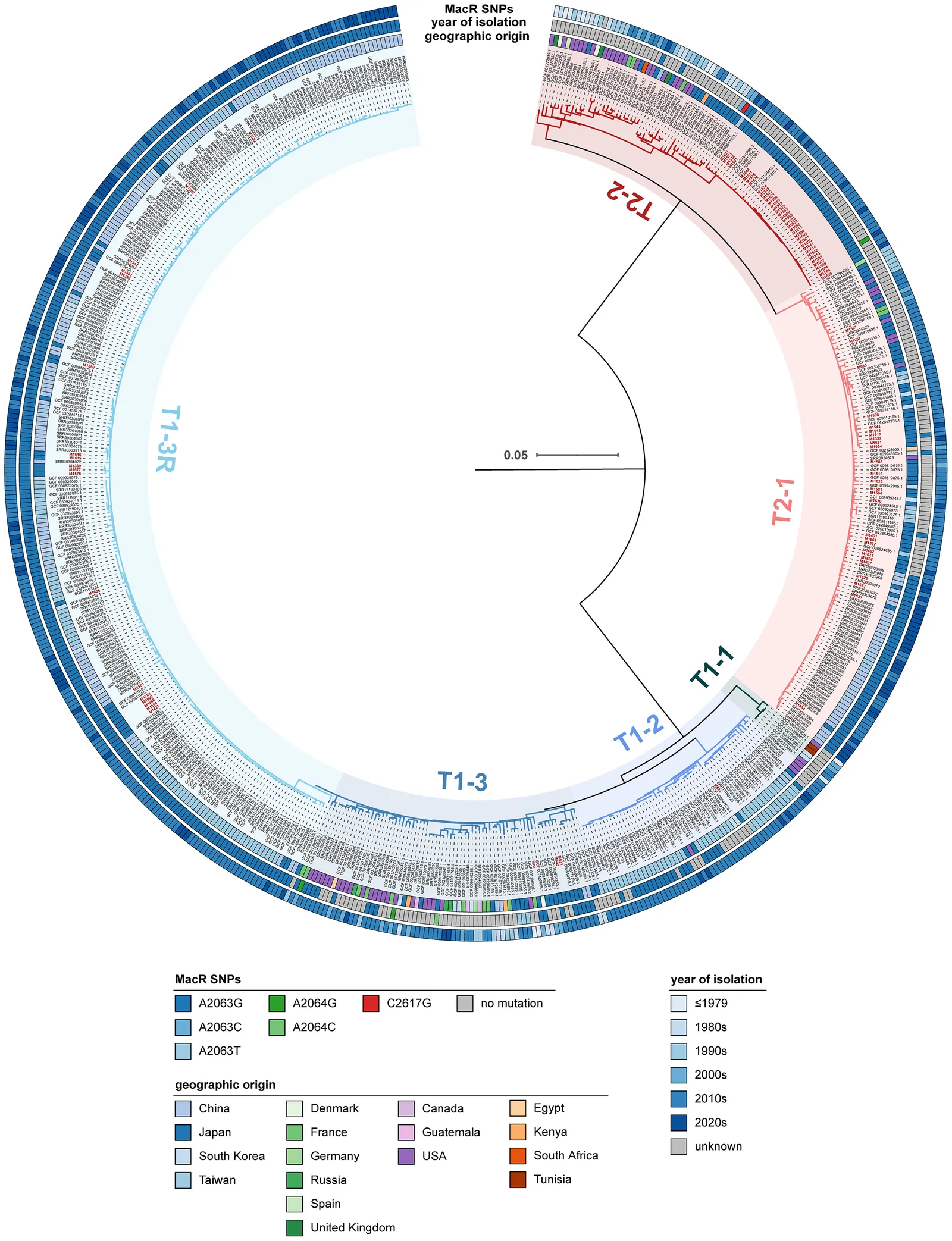
Core genome SNP-based phylogeny of 613 *M. pneumoniae* strains. Maximum-likelihood phylogenetic tree constructed from core genome–derived single-nucleotide polymorphisms (SNPs) identified among 613 *M. pneumoniae* strains, including 86 strains sequenced in this study and 527 strains retrieved from public genome databases. Strains sequenced in the present study are highlighted in red. Clade assignments are indicated by branch colors and shaded background regions, with clade names shown on the tree. Concentric outer rings indicate, from inside to outside, geographic origin, year of isolation (grouped into temporal categories), and macrolide resistance–associated 23S rRNA gene mutations (MacR_SNPs).

To obtain an overall view of the phylogenetic relationships among *M. pneumoniae* strains, including the phylogenetic placement of macrolide-resistant strains, and to characterize clade structure along with the distribution of resistant strains within clades, we constructed a phylogenetic tree based on core genome–derived SNPs (**Figure 3**). Key metadata, including geographic origin, year of isolation, clade assignment, and the presence or absence of macrolide resistance-associated mutations, were overlaid onto the phylogenetic tree. According to the currently accepted classification framework for *M. pneumoniae*, type 1 lineage strains are subdivided into four clades (T1-1, T1-2, T1-3, and T1-3R), whereas type 2 lineage strains are subdivided into two clades (T2–1 and T2-2). Within the dataset, the distribution of clades was uneven. The T1-3R clade was the most prevalent, comprising 43.1% of strains, whereas T1–1 was rare, representing only 1.0% of strains. Other clades, including T1-2, T1-3, T2-1, and T2-2, showed intermediate frequencies (**Supplementary Figure 1**). This skewed clade distribution may reflect the predominance of certain lineages in specific geographic regions, suggesting a potential link between clade composition and geographic origin. The phylogenetic tree further suggested that each clade exhibited characteristic patterns, with apparent differences in the proportion of macrolide-resistant strains and in their geographic distribution.

### 3.4 Relationship between clade composition and geographic distribution

**Figure 4:**
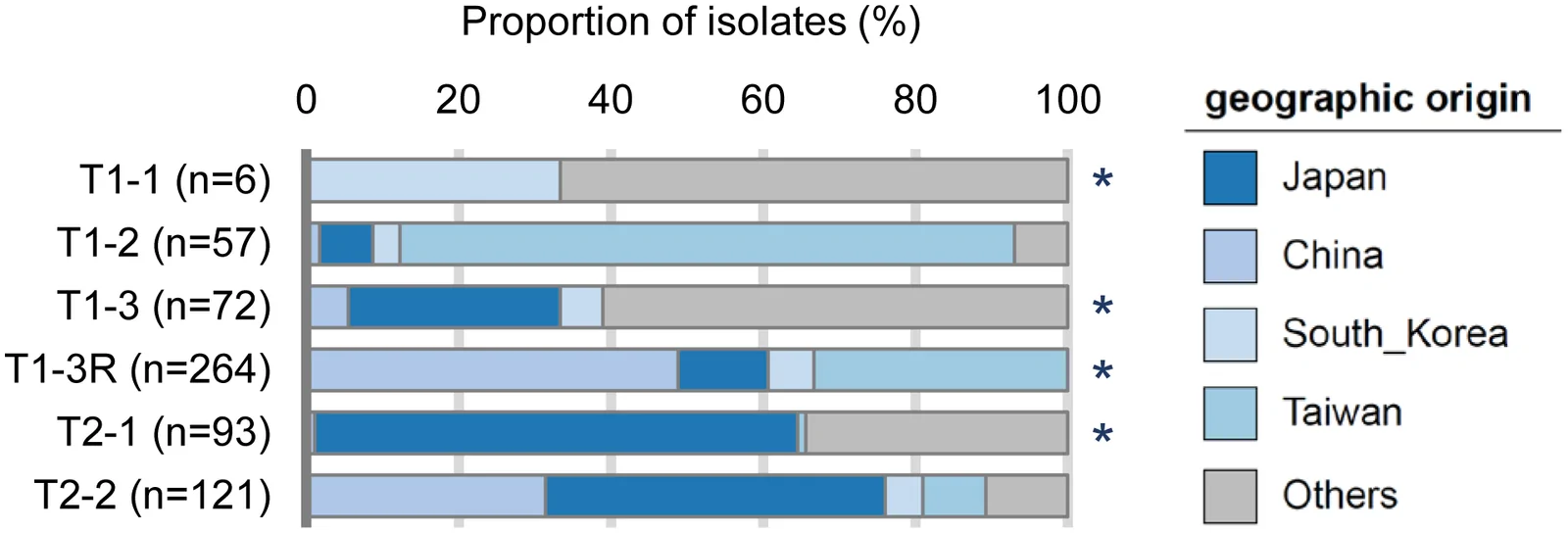
Geographic distribution of strains among clades. The proportions of strains from different geographic origins within each clade are shown as 100% stacked bar charts. The number of strains in each clade is indicated in parentheses. East Asian regions are shown individually, whereas strains from all other regions are grouped together and presented as “Others.” Differences in the proportions of East Asian versus non–East Asian strains among clades were assessed using Fisher’s exact test with false discovery rate (FDR) correction. * indicates FDR-adjusted *P* < 0.05.

The phylogenetic tree suggested potential differences in the geographic distribution among clades. To quantitatively assess these differences, we summarized the proportions of strains from different geographic origins within each clade (**Figure 4**). All strains belonging to T1-3R, a clade predominantly composed of macrolide-resistant strains, were derived from East Asia, with no strains identified from other regions. Similarly, T1–2 and T2–2 showed a tendency toward a higher proportion of strains derived from East Asia, although these differences did not reach statistical significance (T1-2: *P* = 0.067; T2-2: *P* = 0.103). In contrast, clades T1-1, T1-3, and T2–1 were characterized by a higher proportion of strains originating from regions outside East Asia. These findings suggest geographic differences in clade composition among *M. pneumoniae* populations.

### 3.5 Differences in macrolide resistance rates among clades

**Figure 5:**
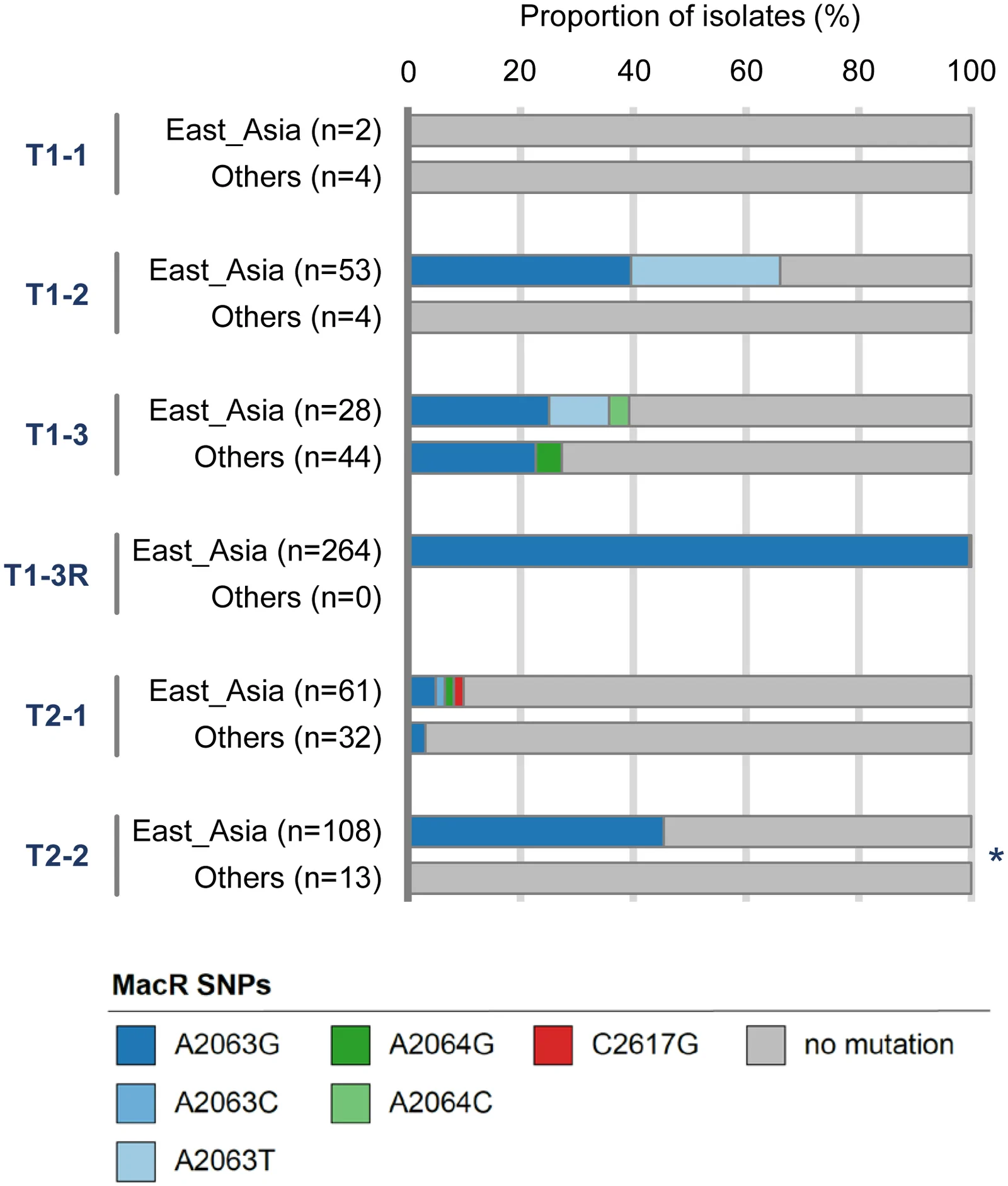
Regional differences in macrolide resistance within clades. The proportions of strains carrying macrolide resistance–associated SNPs in the 23S rRNA gene are shown for isolates from East Asia and non–East Asian geographic origins within each clade as 100% stacked bar charts. The number of strains in each group is indicated in parentheses. In the figure, non–East Asian regions are collectively shown as “Others.” Differences in resistance proportions between East Asian and non–East Asian isolates were assessed for each clade using Fisher’s exact test with false discovery rate (FDR) correction. * indicates FDR-adjusted *P* < 0.05.

The phylogenetic tree also suggested differences in the proportions of macrolide-resistant strains among clades. To quantitatively assess these patterns, we compared MRMP proportions across clades (**Supplementary Figure 2**). As expected, the resistance-associated clade T1-3R was predominantly composed of resistant strains. In addition, clades T1-2, T1-3, and T2–2 also contained substantial proportions of MRMP, each accounting for approximately 40% of strains. These findings raised the possibility that increased proportions of resistant strains within specific clades may be associated with the elevated MRMP isolation rate observed in East Asia. To explore this, we examined whether resistance rates differed between geographic regions within each clade (**Figure 5**). Within clade T2-2, strains from East Asia exhibited a significantly higher resistance rate than those from other regions. Although statistical significance was not reached for T1-2 (*P* = 0.055), a similar regional pattern was observed. Approximately 66% of East Asian strains were macrolide resistant, whereas no resistant strains were detected in other regions. In contrast, no clear regional difference in resistance proportions was observed for clade T1-3. Importantly, both T2–2 and T1–2 are frequently isolated in East Asia, suggesting that the higher prevalence of MRMP in this region cannot be attributed solely to the expansion of the resistance-associated clade T1-3R but may also involve resistant lineages circulating within additional clades.

### 3.6 Phylogenetic network analysis of the dissemination patterns of resistant clones

**Figure 6:**
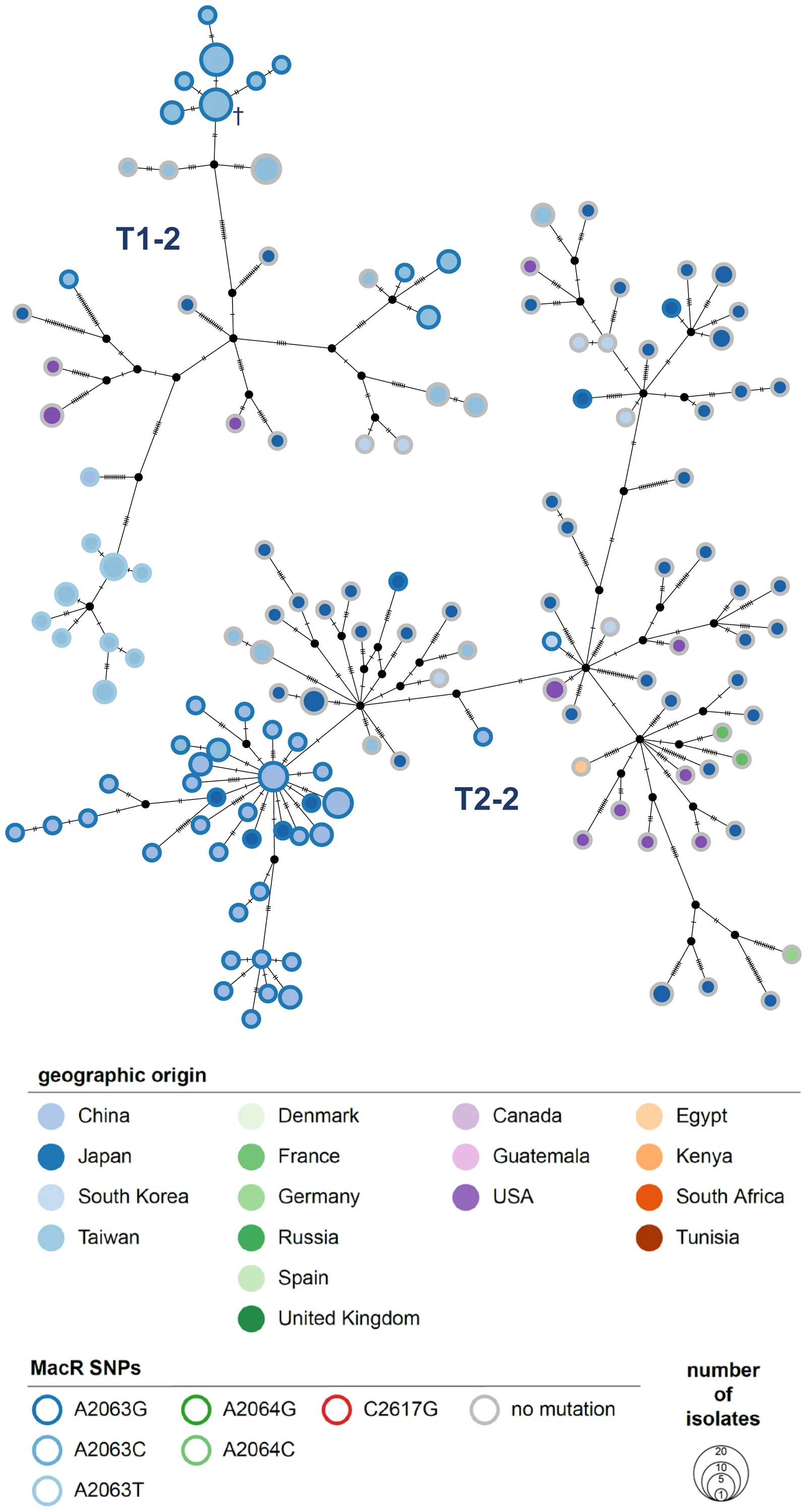
Phylogenetic networks of *M. pneumoniae* isolates in clades T1–2 and T2-2. Phylogenetic networks for clades T1–2 and T2–2 were constructed based on core genome–derived SNPs using POPART v1.7. Each node represents isolates with identical core genome genotypes, and node size is proportional to the number of isolates. Nodes are colored according to geographic origin, and the border color indicates the type of macrolide resistance–associated SNP in the 23S rRNA gene. Connections between nodes represent genetic relationships, and the number of hatch marks on each edge indicates the number of SNP differences. Because the 23S rRNA gene was not included in the core genome alignment, isolates within the same node may differ in resistance-associated SNPs; such cases are indicated by annotations. The network is consistent with regional expansion of resistant clones. † Node contains 5 isolates: 4 carrying A2063G and 1 without resistance-associated mutations. The node border color represents the majority SNP type.

Given the observed differences in resistance proportions and geographic distribution among clades, phylogenetic network analysis was performed to examine the expansion and dissemination patterns of macrolide-resistant lineages and to clarify genetic relationships among closely related strains, thereby delineating clonal structure. Clades T1–2 and T2–2 were selected for analysis based on their relatively high resistance proportions and geographic bias toward East Asia (**Figure 6**). In both clades, MRMP formed clearly defined clusters in the network, composed exclusively of strains from East Asia. These observations are consistent with the emergence of specific resistant clones that subsequently expanded within East Asia. Notably, in clade T2-2, strains belonging to the same resistant cluster were distributed across multiple geographic locations within East Asia, indicating inter-regional transmission within East Asia rather than restriction to a single country or area. Analyses of clades T1–3 and T2-1, which were infrequently isolated in East Asia, revealed only sporadic occurrence of MRMP without evidence of broader clustering, in contrast to that observed in clades T1–2 or T2-2 (**Supplementary Figure 3**). Thus, unlike in East Asia, clonal expansion of resistant strains appears to be limited in non–East Asian regions, potentially contributing to the lower prevalence of macrolide resistance. Furthermore, when focusing on macrolide-susceptible strains, strains from regions outside East Asia included strains that were closely related (within 10 SNPs) to East Asian strains. This finding indicates that *M. pneumoniae* lineages are not restricted to specific geographic regions but instead are distributed more broadly. Collectively, these findings are consistent with the view that the high prevalence of MRMP in East Asia is closely linked to the emergence and regional expansion of resistant clones, whereas the presence of closely related strains across regions indicates the potential for broader geographic dissemination.

## 4 Discussion

In this study, we analyzed genome sequences from a total of 613 *M. pneumoniae* strains, including 86 strains isolated in Japan and 527 strains retrieved from public genome databases. Using phylogenetic tree and network analyses, we characterized the evolutionary relationships among strains across geographic regions, their clade composition, and the distribution of macrolide-resistant isolates. More than 60% of the analyzed strains were identified as MRMP, with the majority harboring the A2063G mutation in the 23S rRNA gene. This finding is generally consistent with previous reports from Japan, China, and other East Asian countries, indicating that our dataset broadly reflects the currently reported epidemiological situation (Kim et al., 2022; Wang et al., 2022; Liu et al., 2024; Wang et al., 2024; Kenri et al., 2020; Wu et al., 2024). Beyond confirming the high prevalence of MRMP, we integrated clade composition, geographic distribution, and macrolide resistance data to characterize the distribution patterns of resistant strains from a population-structure perspective. As a result, we showed that the high prevalence of macrolide resistance in East Asia is not a uniform feature across the entire *M. pneumoniae* population but is instead preferentially associated with specific clades.

In the clade-based analysis, the macrolide-resistant clade T1-3R was predominantly composed of strains originating from East Asia in the present dataset. This observation is consistent with previous reports indicating that the dissemination of T1-3R has contributed substantially to the increasing prevalence of MRMP in East Asia, reaffirming its established epidemiological significance. Analysis of the other clades revealed distinct patterns: T1–2 and T2-2, which are frequently isolated in East Asia, contained resistant isolates with markedly higher resistance rates among East Asian strains than among strains from other regions. In contrast, T1-3, though predominantly distributed outside East Asia, contained a moderate proportion of resistant isolates, with similar MRMP frequencies between East Asian and non–East Asian strains. Meanwhile, T2-1, also mainly distributed outside East Asia, exhibited consistently low proportions of MRMP. Phylogenetic tree and network analyses further demonstrated that, within T1–2 and T2-2, resistant strains were not randomly distributed but instead formed distinct clusters that subsequently expanded across multiple regions in East Asia. These findings suggest that the high prevalence of MRMP in East Asia may be driven less by an increased frequency of resistance mutation emergence, and more by regional differences in the expansion and dissemination patterns of resistant clones. Although the present analyses do not directly infer the number of independent resistance acquisition events, the presence of distinct resistant clusters within multiple clades is consistent with the emergence and subsequent expansion of resistant lineages in different genetic backgrounds.

Although East Asia was characterized overall by a high prevalence of the T1-2, T1-3R and T2–2 clades, notable differences were observed among individual countries. For example, T1–3 and T2-1, which were relatively uncommon in East Asia as a whole, were more frequently detected among Japanese isolates. Likewise, the prevalence of macrolide resistance in Japan was substantially lower than that observed in China despite both countries belonging to the same geographic region. These observations suggest that the population structure of *M. pneumoniae* and the epidemiological dynamics of macrolide-resistant strains may differ considerably among countries within East Asia. Further studies will be required to better understand the evolutionary and epidemiological factors underlying these intra-regional differences.

Multiple factors are likely involved in the region-specific expansion of macrolide-resistant clones observed in East Asia, suggesting the presence of selective pressures that favor their establishment and dissemination. Among these, antimicrobial use is considered a major driver of resistance emergence (Goossens et al., 2005; Abejew et al., 2024; Batenburg et al., 2020). Because *M. pneumoniae* infections are predominantly managed in outpatient settings, particularly among pediatric patients, in whom antibiotics are frequently prescribed, antimicrobial use in these settings may influence the selection and spread of macrolide-resistant strains. Regional differences in prescribing practices could therefore partly explain the observed variation in resistant clone expansion. Previous studies have shown that outpatient antibiotic prescription rates are higher in East Asia than in Europe and North America (Kasse et al., 2024; Youngster et al., 2017; Wushouer et al., 2024; Cao et al., 2023; Iwamoto et al., 2022), potentially creating an environment that favors strains carrying macrolide resistance–associated mutations. In addition, azithromycin use has been suggested to contribute to the emergence and dissemination of MRMP. Although azithromycin is convenient due to its short dosing regimen, its pharmacokinetic properties result in prolonged exposure to sub-inhibitory concentrations, which may facilitate the selection of resistant strains. Reports from China and the United States have indicated an association between azithromycin use and macrolide resistance (Wang et al., 2025a; Yan et al., 2025). Taken together, variations in antimicrobial prescribing practices may contribute to the regional differences in the expansion of resistant clones. Clarifying the factors that promote the establishment and spread of resistant clones will be important for guiding future infection control and antimicrobial stewardship efforts.

As described above, the expansion of macrolide-resistant clones is currently observed predominantly in East Asia. However, in this study, we identified several macrolide-susceptible strains that were genetically closely related (within 10 SNPs) despite being isolated in different geographic regions, including East Asia and other areas. This finding suggests that international dissemination of *M. pneumoniae* strains have already occurred to a certain extent. Therefore, even in regions where the prevalence of macrolide-resistant strains is currently low, the possibility that resistant clones may be introduced and become established in the future cannot be excluded, particularly in association with human mobility and epidemic spread. The observed international genetic relatedness among macrolide-susceptible strains indicates that the regional differences identified in this study may not be static, underscoring the importance of continuous genomic surveillance from a global perspective.

Several limitations should be considered when interpreting the findings of this study. First, because strains with publicly available genome sequences are unevenly distributed with respect to their geographic origins, the analyzed strains were predominantly derived from East Asia, whereas the number of strains from other regions was relatively limited. As a result, some regional comparisons may have lacked sufficient statistical power. In addition, publicly available genomes were not evenly distributed across isolation years, and therefore the observed geographic patterns may have been influenced, at least in part, by temporal and sampling biases. The primary objective of this study was to investigate geographic patterns of macrolide resistance and clade distribution at the population level, and further stratification by isolation period would have substantially reduced sample sizes within several geographic groups, particularly outside East Asia. Accordingly, comparisons between East Asian and non–East Asian regions should be interpreted with caution. Furthermore, because isolates from Europe, the Americas, and Africa were relatively limited in number, the findings of this study primarily reflect the epidemiological situation in East Asia and should not be directly generalized to other regions without further validation using larger and more geographically representative datasets. Second, this study was primarily based on genomic data, and epidemiological factors such as clinical background, disease severity, and antimicrobial usage patterns could not be directly evaluated. Therefore, although potential factors related to the expansion of macrolide-resistant clones are discussed above, these interpretations should be regarded cautiously rather than as definitive conclusions. Further studies incorporating detailed epidemiological and clinical data will be required to validate these possibilities.

In conclusion, this study demonstrates that the high prevalence of MRMP is not explained solely by the expansion of a single resistant clade. Instead, our findings suggest that the dissemination of resistant clones across multiple regionally predominant clades has contributed to the formation of the current resistance landscape. The presence of distinct resistant clusters within multiple clades is consistent with the emergence and subsequent expansion of resistant lineages in different genetic backgrounds. The observation that macrolide resistance rates vary markedly by geographic region even within the same clade highlights the importance of considering both clade structure and regional transmission dynamics when interpreting resistance trends. Although the present dataset was dominated by East Asian strains, our findings provide insight into the mechanisms underlying the exceptionally high prevalence of MRMP in this region. Additional genomic data from underrepresented regions will be important for clarifying whether regional differences in resistance prevalence are associated with differences in clonal expansion dynamics, population structure, or other epidemiological factors. These insights provide a framework for understanding the processes underlying the acquisition and spread of macrolide resistance and underscore the value of continued genome-based epidemiological surveillance.

## Data Availability

The datasets generated and/or analyzed during the current study are available in the DDBJ BioProject database under accession number PRJDB42251.
