# Supplementary material for: Population structure reveals clade- and region-specific dissemination of macrolide-resistant Mycoplasma pneumoniae
Source: Front Cell Infect Microbiol. 2026 Aug 10;16:1891292. doi: 10.3389/fcimb.2026.1891292 (PMC13500242; doi:10.3389/fcimb.2026.1891292)
Supplement: Supplementary file 1 [file Image1.pdf]

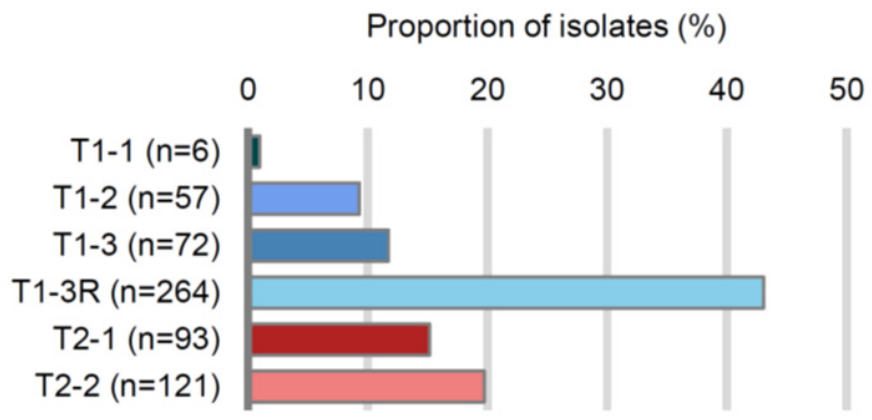


**Supplementary Figure S1. Clade composition of the 613 *M. pneumoniae* strains.**

The proportional distribution of clades among all analyzed strains is shown as a percentage bar chart. The absolute number of strains in each clade is indicated in parentheses.

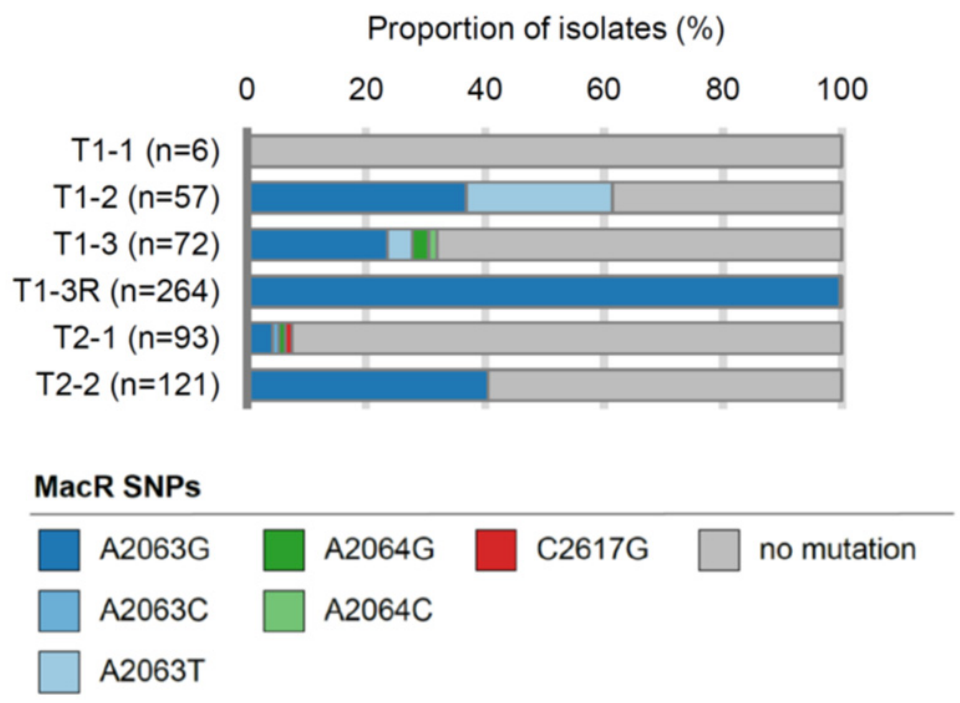


**Supplementary Figure S2. Proportion of macrolide resistance–associated 23S rRNA mutations across clades.**

The proportions of strains carrying macrolide resistance–associated SNPs in the 23S rRNA gene within each clade are shown as 100% stacked bar charts. The number of strains in each clade is indicated in parentheses.

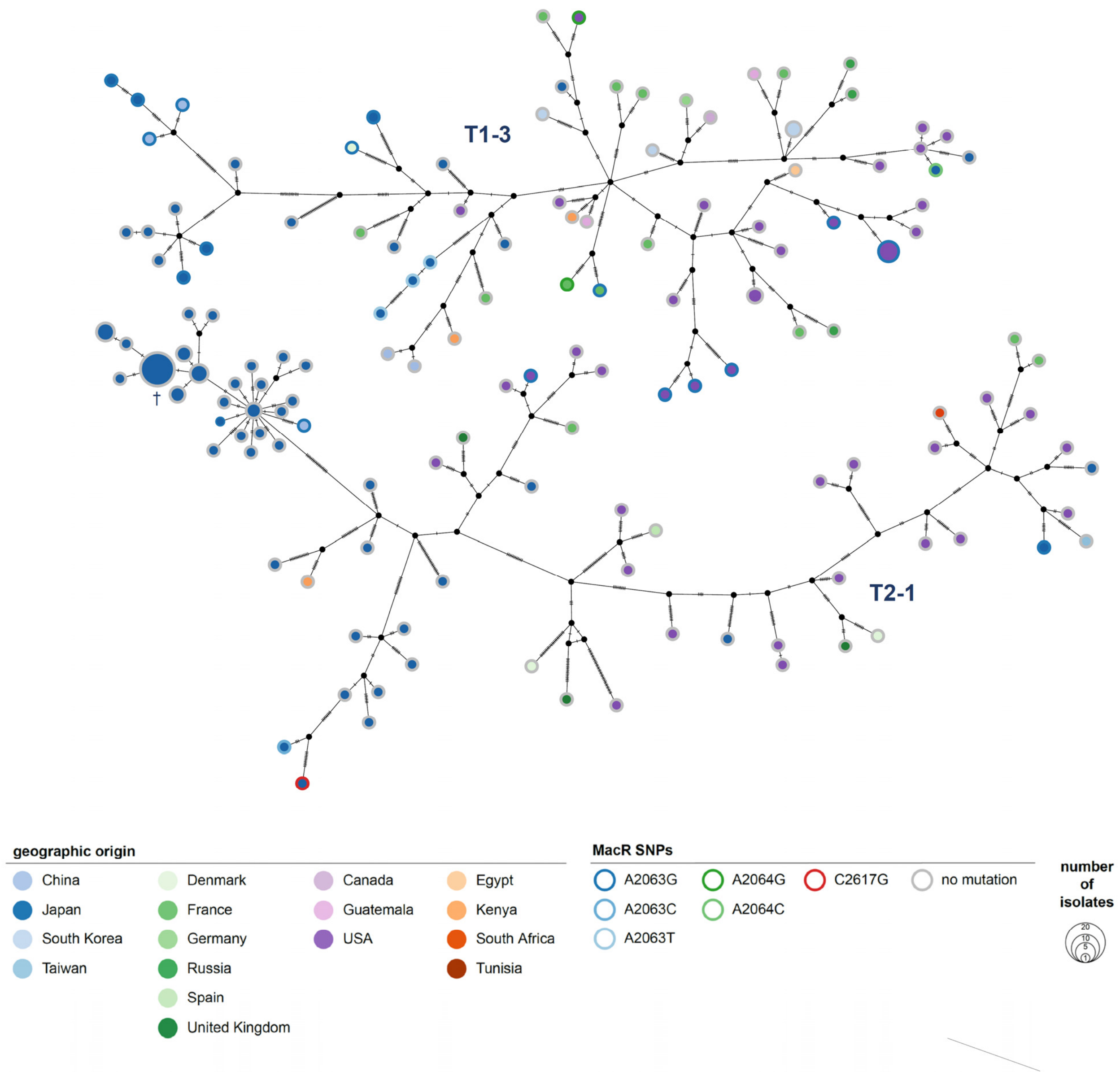


**Supplementary Figure S3. Phylogenetic networks of *M. pneumoniae* isolates in clades T1-3 and T2-1.**

Phylogenetic networks for clades T1-3 and T2-1 were constructed based on core genome SNPs using the PopART program. Each node corresponds to a group of isolates sharing an identical core genome genotype, and the size of each node reflects the number of isolates. Node colors indicate geographic origin, while border colors denote the type of macrolide resistance–associated SNP in the 23S rRNA gene. Links between nodes represent genetic relationships, with the number of hatch marks indicating SNP differences. As the 23S rRNA gene was excluded from the core genome alignment, isolates within the same node may differ in resistance-associated SNPs; such instances are annotated accordingly. Unlike clades T1-2 and T2-2, no clear clustering of resistant isolates was observed.

† Node contains 13 isolates: 1 carrying A2064G and 12 without resistance-associated mutations. The node border color represents the majority SNP type.
